# Stoichiometry for α-bungarotoxin block of α7 acetylcholine receptors

**DOI:** 10.1038/ncomms9057

**Published:** 2015-08-18

**Authors:** Corrie J. B. daCosta, Chris R. Free, Steven M. Sine

**Affiliations:** 1 Department of Physiology and Biomedical Engineering, Mayo Clinic College of Medicine, 200 First Street SW, Rochester, Minnesota 55905, USA; 2 Department of Neurology, Mayo Clinic College of Medicine, 200 First Street SW, Rochester, Minnesota 55905, USA

## Abstract

α-Bungarotoxin (α-Btx) binds to the five agonist binding sites on the homopentameric α7-acetylcholine receptor, yet the number of bound α-Btx molecules required to prevent agonist-induced channel opening remains unknown. To determine the stoichiometry for α-Btx blockade, we generate receptors comprised of wild-type and α-Btx-resistant subunits, tag one of the subunit types with conductance mutations to report subunit stoichiometry, and following incubation with α-Btx, monitor opening of individual receptor channels with defined subunit stoichiometry. We find that a single α-Btx-sensitive subunit confers nearly maximal suppression of channel opening, despite four binding sites remaining unoccupied by α-Btx and accessible to the agonist. Given structural evidence that α-Btx locks the agonist binding site in an inactive conformation, we conclude that the dominant mechanism of antagonism is non-competitive, originating from conformational arrest of the binding sites, and that the five α7 subunits are interdependent and maintain conformational symmetry in the open channel state.

## Abstract (web summary)

Since their discovery more than fifty years ago, α-neurotoxins have been used to study acetylcholine receptor-coupled ion channels. Here, daCosta *et al*. find that toxin binding to a single site of the pentameric α7 receptor blocks function, suggesting the five binding sites are interdependent and the toxin arrests the sites in the inactive conformation.

## Graphical abstract

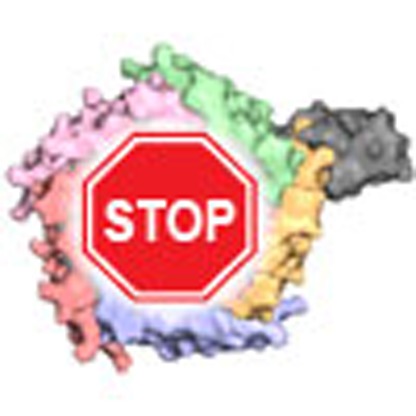

α-Neurotoxins from the venom of snakes in the family Elapidae are notorious for producing long-lived neuromuscular blockade^1^. They are basic peptides comprised of 60–70 amino acids that, through intra-molecular disulfide bonds, form three fingers that extend from a globular base^2^. Binding of α-neurotoxin to the acetylcholine receptor (AChR) at the motor synapse is mutually exclusive toward binding of small molecule agonists and antagonists^3^, and X-ray crystallographic studies show that α-neurotoxins, agonists and antagonists contact overlapping regions at the ligand-binding site^4^,^5^,^6^,^7^,^8^,^9^. The overall physiological, pharmacological and structural studies suggest that α-neurotoxin blockade operates through a competitive mechanism.

Members of the nicotinic AChR family contain from two to five agonist binding sites. Studies of the muscle AChR, which contains two agonist binding sites, showed that α-neurotoxin occupancy of one site rendered the agonist-induced response undetectable^10^,^11^. Those observations harmonized with subsequent single channel measurements showing that AChRs occupied by one agonist opened the channel with much lower efficiency than those occupied by two agonists^12^. Thus consistent with a competitive mechanism, occupancy of one site by α-neurotoxin prevented binding of a second agonist required for efficient channel opening. However, given subsequent structural data^4^,^7^, an alternative mechanism is equally plausible: α-neurotoxin occupancy locks the binding site in an inactive conformation and conformational arrest of that site prevents channel opening.

To distinguish between competitive and conformational arrest mechanisms, we devise a strategy to assess α-neurotoxin occupancy of the α7 AChR simultaneously with agonist-induced channel opening. The α7 AChR contains five identical subunits and binds the α-neurotoxin, α-Bungarotoxin (α-Btx), at each of its five agonist binding sites (Fig. 1)^13^, thus offering the maximum number of sites to study the occupancy-channel opening relationship. Taking advantage of a mutant subunit that confers α-Btx resistance, we generate receptors comprised of wild-type and α-Btx-resistant subunits, and tag one of the subunit types with conductance mutations to report subunit stoichiometry^14^,^15^,^16^,^17^,^18^. Following incubation with α-Btx, we make patch clamp recordings to monitor opening of individual α7 AChRs with defined numbers of bound α-Btx molecules. The findings not only distinguish between the two mechanisms of α-neurotoxin blockade, but they also suggest the five subunits are interdependent and maintain conformational symmetry in the open channel state.

## Results

### Experimental strategy

To determine the number of α-Btx molecules that block opening of individual α7-receptor channels, we devised the following experimental strategy: generate an α7 subunit that prevents α-Btx binding yet still allows activation by agonist; generate the low conductance form of the wild-type α7 subunit; co-transfect HEK cells with complementary DNAs (cDNAs) encoding the two types of subunits to form pentameric receptors with variable subunit stoichiometry; record single channel currents before and after incubation with α-Btx; measure the current amplitude of each channel opening event to determine the subunit stoichiometry^17^,^18^; following incubation with α-Btx, infer the number of bound α-Btx molecules from the subunit stoichiometry.

### A functional α-Btx-resistant mutant

**Figure 1:**
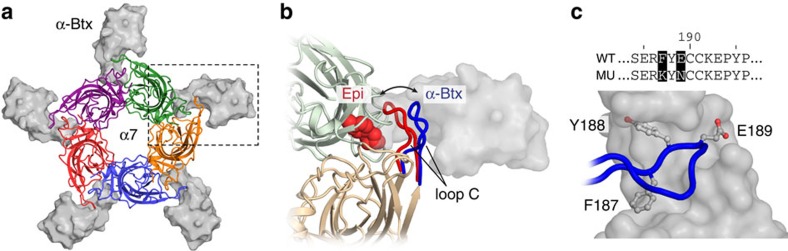
Structural details of α-Btx binding to α7. (**a**) Complex between the α7-acetylcholine receptor ligand binding domain chimera (α7; ribbons, where each subunit is a different colour) and α-Btx (grey surfaces; PDB: 4HQP). (**b**) Close up view of the boxed region in ‘a’ showing α7 in complex with the agonist epibatidine (Epi, red spheres; PDB: 3SQ6) and α-Btx (grey surface). The conformations of ‘loop C’ in both the Epi-α7 (red) and α-Btx-α7 (blue) complexes are overlaid to show how the toxin locks loop C in an extended conformation. With the exception of the epibatidine (red spheres) and the Epi-loop C (red), the structures depicted are from the α-Btx-α7 complex (PDB: 4HQP). (**c**) Sequence alignment of loop C residues in wild-type α7 (WT) and the toxin-resistant mutant (MU). Shown below is a close up of the interaction between WT-loop C (blue) and α-Btx (grey surface; PDB: 4HQP), where the wild-type side chains of the residues substituted in the mutant, which flank a canonical Tyrosine residue (Y188), are shown in a ball and stick representation.

To generate functional, α-Btx-resistant α7 receptors, we considered sequence differences among naturally occurring nicotinic AChR α-subunits, such as the α1-subunits from the snake and mongoose^19^,^20^ and the α2-α4 subunits from heteromeric neuronal AChRs^21^,^22^. All of these subunits harbour subtle changes in a structure known as loop C, a β-hairpin at the entrance of the AChR ligand binding site, and a major site for interaction with α-Btx (Fig. 1). We chose to mimic sequence differences in loop C from the neuronal α3 subunit, a strategy that was successful in generating a functional, α-Btx-resistant α7/5-HT_3A_ receptor^23^, and mutated the pair of residues flanking a canonical tyrosine residue within loop C to their α3 counterparts (Fig. 1).

**Figure 2:**
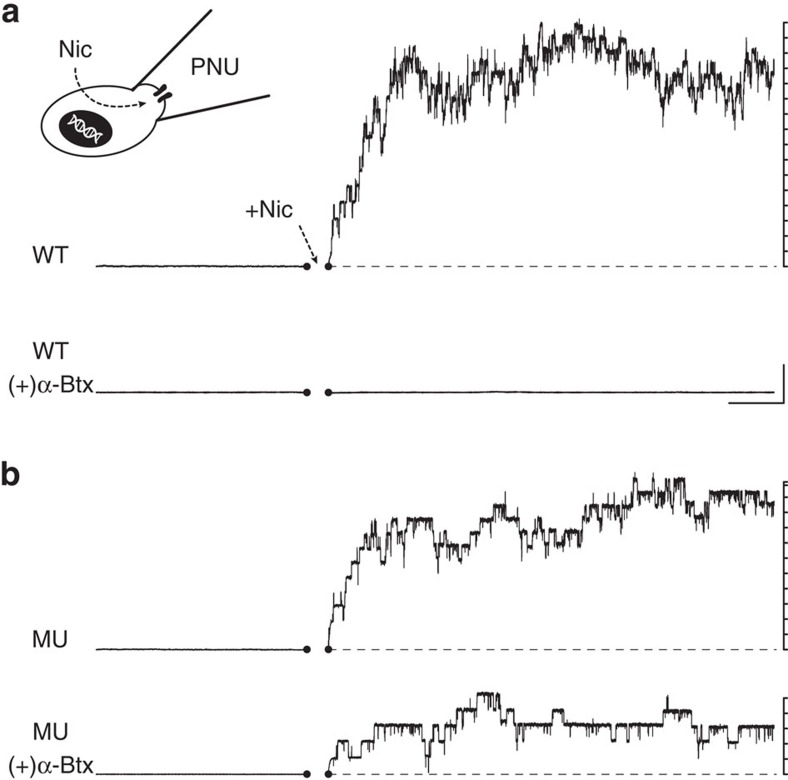
A functional α-Btx-resistant α7 mutant. Difference in toxin sensitivity for homopentameric receptors formed from (**a**) wild-type (WT, top two traces) and (**b**) mutant α-Btx-resistant subunits mutant ((MU), bottom two traces). After recording a stable baseline free of single channel openings (that is, no agonist present), nicotine (100 μM final concentration) was added to the bath solution surrounding each patch (arrow). For the lower traces, in both ‘a’ and ‘b’, cells were incubated with 50 nM α-Btx for 20 min before forming a cell-attached patch. A concentration of 10 μM PNU was included in all patch pipettes to prolong agonist-activated openings and facilitate their visualization (see Methods). Note that each recording was obtained from a different membrane patch, on a different cell, so the magnitude of the evoked currents is not comparable between recordings. Traces are filtered at 100 Hz, each increment of the vertical axes (right) indicates 10 pA, and scale bar, 5 s, 25 pA.

To confirm that receptors comprised of α-Btx-resistant subunits fulfilled the dual requirements of α-Btx resistance and agonist responsiveness, we transfected cDNAs encoding either wild-type α7 or α-Btx-resistant subunits into HEK cells, and incubated the cells with either buffer alone or buffer containing 50 nM α-Btx. We then formed a cell-attached patch with the potentiator PNU-120596 (PNU) in the pipette solution to prolong the inherently brief α7-channel openings on subsequent addition of agonist^24^,^25^. After recording a baseline free of channel openings, we added the membrane-permeable agonist nicotine to the external solution. For cells expressing either wild-type or α-Btx-resistant receptors, addition of nicotine elicits single channel currents that sum in a staircase manner, indicating robust receptor activation by agonist (Fig. 2). Following incubation with α-Btx, channel opening of wild-type α7 receptors is completely blocked, whereas channel opening of α-Btx-resistant receptors still occurs. Thus, receptors comprised of α-Btx-resistant subunits respond to agonist but are not blocked by α-Btx.

**Figure 3:**
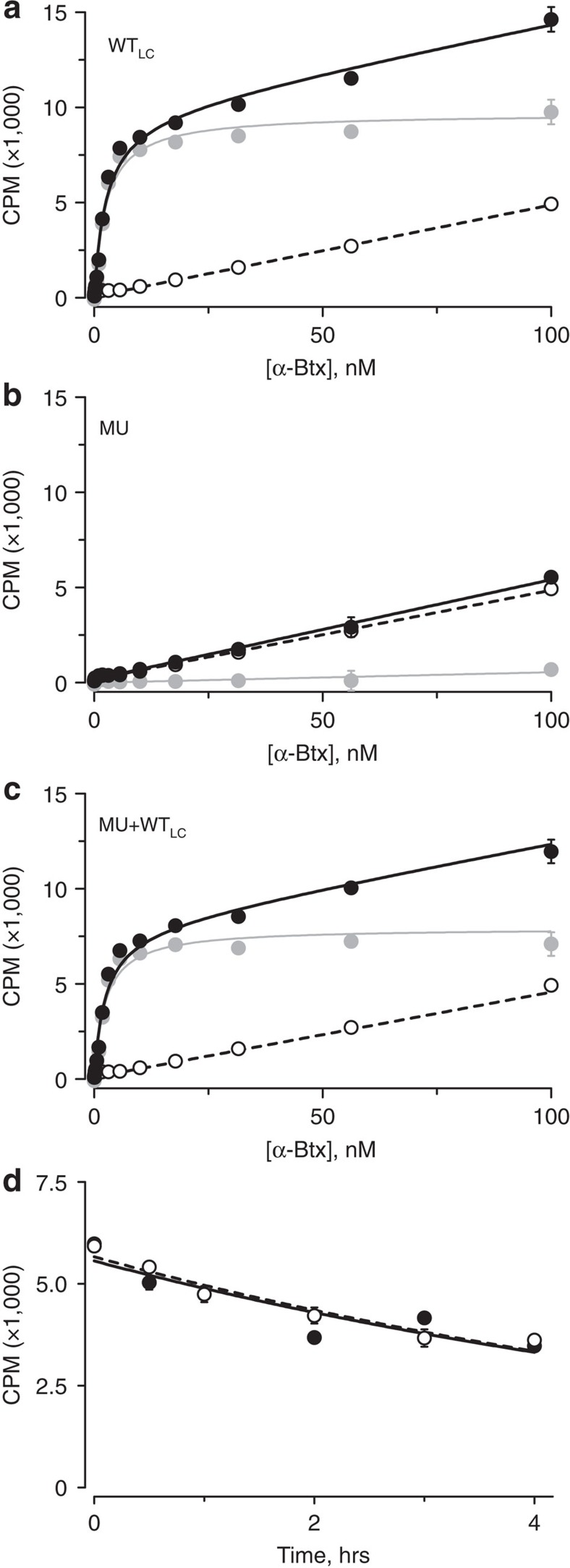
Steady state α-Btx binding measurements. Radiolabeled [^125^I]-α-Btx binding to cells expressing (**a**) wild-type, low-conductance (WT_LC_) subunits, (**b**) mutant subunits (MU), or (**c**) mixtures of MU and WT_LC_ subunits (1:4, MU:WT_LC_). In each case, total binding (closed circles, solid line), non-specific binding (open circles and dashed line), as well as specific binding (grey circles and line) are shown. Non-specific binding was determined from cells transfected with the cDNA encoding the human muscle AChR β1-subunit, which does not form α-Btx binding sites. The apparent K_d_s for cells expressing WT_LC_ subunits, and mixtures of MU and WT_LC_ subunits are 2.7 nM (2.26–3.14 nM) and 2.4 nM (1.90–2.84 nM), respectively, (95% confidence limits), where *n*=3 and the error bars represent one s.d. of the mean. (**d**) Time course of [^125^I]-α-Btx dissociation from cells expressing WT_LC_ (closed circles and solid line), or mixtures of MU and WT_LC_ subunits (1:4, MU:WT_LC_; open circles and dashed line). The dissociation half-life for cells expressing WT_LC_ subunits and mixtures of MU and WT_LC_ subunits are 322.6 min (239.4–494.3 min) and 316.7 min (258.0–410.0 min), respectively, (95% confidence limits), where *n*=2 and error bars are one s.d. of the mean.

Another requirement is that for receptors containing both α-Btx-resistant and wild-type subunits, α-Btx binds normally to sites formed from wild-type subunits. Thus, we transfected HEK cells with cDNAs encoding the wild-type α7 subunit, the α-Btx-resistant subunit or a mixture of the two. We then incubated intact cells with increasing concentrations of radio-labelled α-Btx, and measured the amount of bound radio-ligand under steady state conditions. For wild-type α7, the total binding of α-Btx increases abruptly at low concentrations, but as the concentration is increased, binding increases more gradually and linearly (Fig. 3). To determine non-specific binding, we measured binding of α-Btx to cells transfected with the human-muscle AChR β1-subunit, which does not form α-Btx-binding sites, and observed only the gradual and linear increase in binding over the range of α-Btx concentrations. The difference between total and non-specific binding reveals that binding of α-Btx to cells expressing wild-type α7 receptors is saturable and the dissociation constant is ∼3 nM. By contrast, for cells expressing α-Btx-resistant receptors, binding of α-Btx is very similar to that for cells transfected with the AChR β1-subunit (Fig. 3). Thus, specific binding of α-Btx to receptors containing α-Btx-resistant subunits is negligible over the concentration range tested. For cells transfected with both wild-type and α-Btx-resistant subunits, both the concentration dependence of α-Btx binding and the rate at which bound α-Btx dissociates mimic those observed for cells transfected with wild-type α7 subunits alone (Fig. 3). Thus, for receptors containing both α-Btx-resistant and wild-type subunits, α-Btx binds normally to sites formed from wild-type subunits.

### Electrical fingerprinting

To determine the number of bound α-Btx molecules required to block agonist-induced single channel currents, we employed our previously described electrical fingerprinting method^15^,^16^,^17^,^18^. The method relies on co-assembly of wild-type and mutant subunits, and in one of the subunit types, substitution of arginine for polar or anionic residues in cytoplasmic portals to alter the unitary current amplitude^26^. Previously, following co-assembly of normal and low conductance α7 subunits, patch clamp recordings revealed five distinct current amplitudes, corresponding to pentameric receptors with zero to four low conductance subunits; receptors with five low conductance subunits were not observed, presumably owing to their low current amplitude^17^,^18^. However, in the course of testing different ionic conditions, we found that removal of calcium unmasked small but detectable unitary currents from receptors comprised solely of low conductance subunits. Thus in the following experiments, we used a calcium-free pipette solution to allow identification of all six possible subunit combinations based on current amplitude.

**Figure 4:**
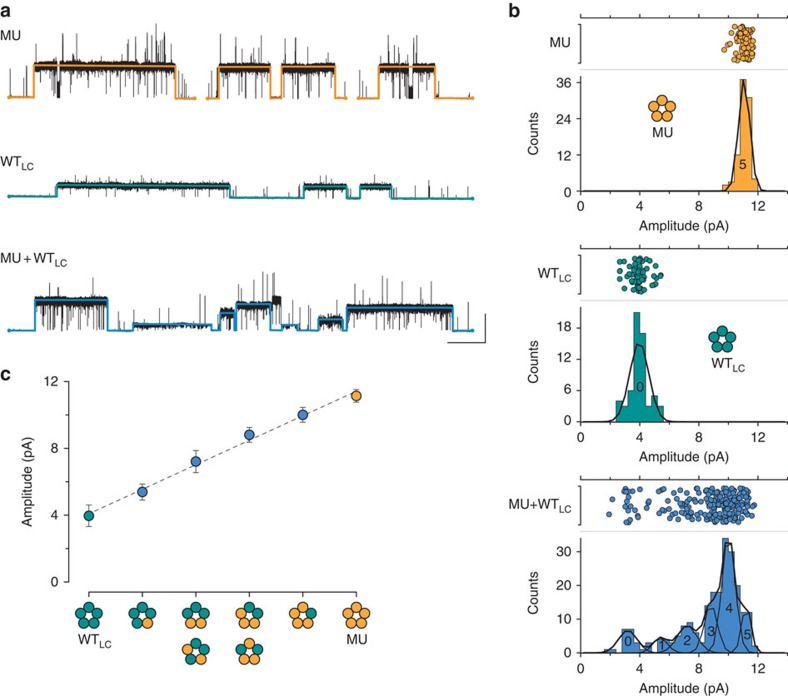
Electrical fingerprinting to determine subunit composition from single channel amplitudes. (**a**) Mutant α-Btx-resistant subunits produce channel openings with a uniform large current amplitude (MU, top), while wild-type, low conductance subunits (WT_LC_) produce openings with a uniform small current amplitude (middle trace). Co-expressing MU subunits with WT_LC_ subunits (1:4; mutant:WT_LC_) gives rise to receptors with different subunit stoichiometry and channel openings with a distribution of amplitudes (bottom trace). For each trace, the detected amplitude for individual clusters of openings, corresponding to a single channel, is overlaid (see Methods for detection criteria; open is up; scale bar, 10 s, 10 pA). (**b**) Event-based amplitude histograms for pooled clusters of openings from mutant subunits (top; 7 recordings and 89 clusters), WT_LC_ subunits (middle; 7 recordings and 62 clusters), and mixtures of mutant and WT_LC_ subunits (bottom; 21 recordings and 225 clusters, pooled from 1:4 and 1:3 cDNA ratios). Amplitude scatter plots corresponding to the openings are also shown above each histogram. Histograms have been fit with a sum of Gaussian components, corresponding to the amplitude classes used to determine (**c**) the relationship between single-channel amplitude and the stoichiometry of MU and WT_LC_ subunits. For each histogram in ‘b’, the Gaussian components are labelled ‘0-5’ according to the presumed number of incorporated mutant subunits. The mean amplitude and s.d. (σ) of each component was used to determine the amplitudes and associated error bars (1σ) used for the plot in ‘c’. The amplitudes for the MU and WT_LC_ classes (yellow and green points) were determined from the corresponding MU and WT_LC_ histograms, whereas for the channels with mixed stoichiometry (blue points) the amplitudes were from the corresponding components in the MU+WT_LC_ histogram.

For cells transfected with cDNA encoding the α-Btx-resistant, normal conductance subunit, patch clamp recordings reveal long-lived single channel openings with large and uniform current amplitudes (Fig. 4). The pipette solution contained ACh and the potentiator PNU, which prolongs channel openings to allow accurate determination of current amplitude, a requirement for electrical fingerprinting. PNU does not affect the ability of α-Btx to bind to α7 receptors^25^ or block channel opening (Fig. 2), likely because it binds within an inter-helical transmembrane cavity^27^. Furthermore, rather than promoting channel opening, PNU dramatically slows the onset of desensitization^25^, which develops from the open channel state^28^. Owing to the submaximal concentration of PNU (1 μM), brief un-potentiated channel openings, resolved only partially at the chosen filter bandwidth, also occur throughout the recordings. For cells transfected with cDNAs encoding the wild-type α7, low conductance subunit, patch clamp recordings again reveal long-lived single channel openings, but their current amplitude, though uniform, is much smaller (Fig. 4). The two diverging current amplitudes represent the maximum and minimum baselines with which to compare receptors formed from combinations of the two types of subunits.

We then co-transfected HEK cells with cDNAs encoding both the α-Btx resistant, normal conductance subunit and the wild-type, low conductance subunit and made patch clamp recordings. The recordings again reveal long-lived single channel openings, but the openings exhibit several different current amplitudes (Fig. 4). As expected from a pentameric assembly of the two types of subunits, the distribution of current amplitudes contains six Gaussian components, corresponding to all possible subunit combinations. Furthermore, the decrease in amplitude between successive components is regular^15^,^16^,^17^,^18^, suggesting that the single-channel amplitude is determined by the number of incorporated low conductance subunits (Fig. 4). Thus, for each channel opening episode, the current amplitude registers the stoichiometry of high- and low-conductance subunits.

Although the two types of subunits co-assembled to form functional receptors, we found that the partitioning of the different current amplitudes did not follow a simple binomial distribution based on the ratio of transfected cDNAs. For example, a 1:1 transfection ratio yielded mainly channel openings with the largest current amplitude, corresponding to receptors with five α-Btx-resistant, normal conductance subunits. The major cause appeared to be a difference in both the time course and level of expression of the two types of receptor subunits. Thus, to obtain the range of current amplitudes in Fig. 4, we transfected the cells with a three- to four-fold molar excess of cDNA encoding the wild-type, low conductance subunit.

### Stoichiometry of α-Btx block

**Figure 5:**
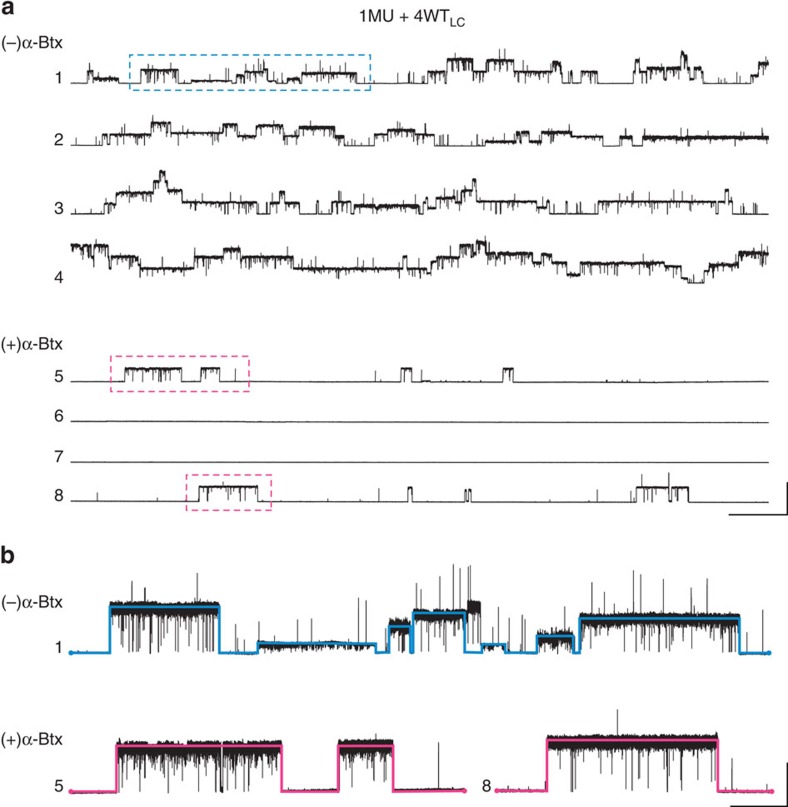
α-Btx inhibition of α7 receptors comprised of both α-Btx-resistant and wild-type subunits. (**a**) Co-expression of mutant α-Btx-resistant subunits with α-Btx-sensitive wild-type, low-conductance subunits (1:4; MU:WT_LC_) gives rise to active patches with multiple levels of superimposed single channel openings (patches 1–4, top). In contrast, when cells that have been transfected by the same mixture are incubated with 50 nM α-Btx, patches have dramatically reduced single channel activity (patches 5–8, bottom). Each sweep represents six uninterrupted minutes of recording from a different patch. Traces are filtered at a bandwidth of 100 Hz; open is up; scale bar, 30 s, 25 pA. (**b**) Enlarged views of the boxed regions in ‘a’, showing that when cells are not pre-incubated with α-Btx, single channel openings are of variable amplitudes and correspond to channels with different numbers of incorporated WT_LC_ subunits (top trace). When cells are incubated with 50 nM α-Btx the infrequent single channel openings are always of large amplitude, corresponding to channels with no, or few, incorporated WT_LC_ subunits. Traces in ‘b’ are filtered at a bandwidth of 1 kHz; open is up; scale bar, 10 s, 10 pA.

To determine the stoichiometry for α-Btx block of α7 channel opening, we co-transfected cells with the subunit cDNAs in ratios of 1:4 and 1:3 (α-Btx-resistant, normal conductance to wild-type and low conductance) and recorded agonist-induced channel openings without and with pre-incubation with α-Btx; a concentration of 50 nM α-Btx was chosen to maximize occupancy of the wild-type binding sites and minimize occupancy of the α-Btx-resistant sites. We first describe results from the 1:4 transfection ratio, which gave the greatest diversity of current amplitudes, and thus the broadest range of subunit stoichiometry. In the absence of α-Btx, patch clamp recordings revealed frequent channel openings, which often superimposed, and the unitary current amplitudes spanned a wide range (Fig. 5). However, after incubation with α-Btx, the frequency of channel opening was greatly suppressed and many patches (13 of 21) exhibited no openings at all (Fig. 5). Equally important, only openings with large current amplitude were present. Thus, in addition to blocking the vast majority of channel openings, α-Btx narrows the distribution of current amplitudes, indicating block depends on the subunit stoichiometry.

**Figure 6:**
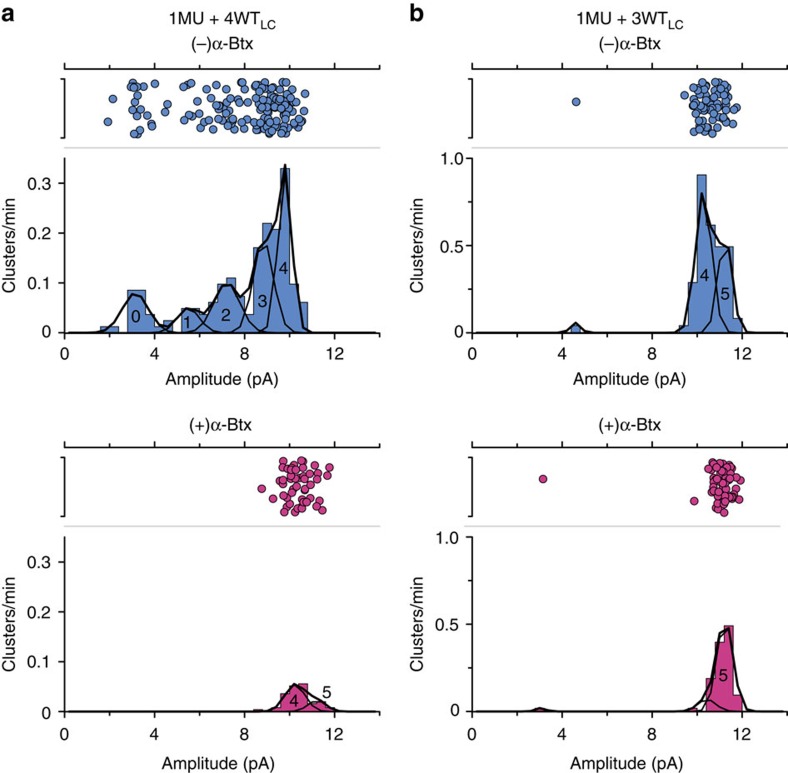
Stoichiometry of α-Btx inhibition of α7. Amplitude histograms, where the number of events in each bin has been scaled to their relative frequency (see Methods), for pooled clusters of openings from cells co-expressing mixtures of mutant (MU) and wild-type, low conductance (WT_LC_) subunits at cDNA ratios of (**a**) 1:4; MU:WT_LC_ and (**b**) 1:3; mutant:WT_LC_. In each case, the top histogram represents the distribution and frequency of openings with a given amplitude when cells are not incubated with α-Btx. When cells transfected in parallel with the same cDNA mixture, are incubated with 50 nM α-Btx, the amplitude distributions change, reflecting relative inhibition by α-Btx (bottom histograms). Histograms have been fit with a sum of Gaussian components, which are labelled ‘0-5’ corresponding to the number of incorporated MU subunits. For each histogram, the number of recordings, observed clusters and total recording time are as follows: A, top, 13 recordings, 153 clusters and 174.7 min; A, bottom, 21 recordings, 50 clusters and 396.6 min; B, top, 8 recordings, 72 clusters, 84.7 min; B, bottom, 7 recordings, 64 clusters and 115.7 min.

To quantify the extent of α-Btx block of receptors with different subunit stoichiometry, we constructed histograms of current amplitudes (Fig. 6). To facilitate comparison between different experimental conditions, the number of clusters in each amplitude bin is expressed relative to both the total number of clusters and the total recording time for each condition (see Methods). For the 1:4 transfection ratio, in the absence of α-Btx, the amplitude histogram contains five of the six possible amplitude classes; openings from the largest amplitude class, corresponding to receptors with five α-Btx-resistant, normal conductance subunits, were not evident as a distinct component (Fig. 6). After incubation with α-Btx, the frequency of clusters of all amplitudes is dramatically reduced and only clusters from receptors with zero or one α-Btx-sensitive subunits were present (Fig. 6). For receptors with one α-Btx-sensitive subunit, the extent of reduction is ∼85%, which approaches the 95% reduction expected if a single occupancy prevented channel opening and occupancy by 50 nM α-Btx was 95%. Thus for the 1:4 transfection ratio in the presence of α-Btx, receptors with more than one α-Btx-sensitive, low conductance subunit are completely blocked and receptors with one α-Btx-sensitive subunit open very rarely.

For the 1:3 ratio, in the absence of α-Btx, the majority of channel openings originated from receptors with either zero or one α-Btx-sensitive, low conductance subunit and the two amplitude classes occurred with a similar frequency (Fig. 6). After incubation with α-Btx, however, virtually all channel openings originated from receptors with five α-Btx-resistant, normal conductance subunits (Fig. 6). Furthermore, the frequency of these openings was essentially the same as that in the absence of α-Btx, which would be expected if the number of α-Btx-resistant receptors was equivalent under the two conditions, and the number of patches was sufficient to minimize patch-to-patch variability in the number of α-Btx-resistant receptors. Furthermore, by achieving a population enriched in receptors with zero or one wild-type, low conductance subunit, we find that α-Btx blocks the vast majority of receptors with a single α-Btx-sensitive site, despite the presence of four sites that remain accessible to the agonist.

## Discussion

We employed an electrical fingerprinting approach to determine the stoichiometry for α-Btx block of α7-AChR channel opening. The results reveal that α-Btx completely blocks receptors with more than one α-Btx-sensitive subunit, and greatly suppresses receptors with just one α-Btx-sensitive subunit. These observations are striking because with one or two α-Btx molecules bound, multiple sites remain unoccupied and available to the agonist, yet in the absence of α-Btx, agonist occupancy of a single site elicits robust channel openings with normal durations^18^. The collective structural and single channel biophysical studies suggest that α-Btx immobilizes the site to which it is bound, which in turn, either alters the interaction between agonist and the remaining sites, interferes with coupling of agonist binding to channel opening, or induces a non-native conformation refractory to channel opening. Thus, although snake α-neurotoxins compete against agonist binding, their dominant mechanism of antagonism is non-competitive, originating from conformational arrest of the binding sites.

Previous studies showed that α-Btx blocked spontaneous channel opening by both the muscle AChR^29^, and an α7 receptor with mutations at the 9’ position of the second transmembrane domain^30^. In common with a conformational arrest mechanism, α-Btx block of spontaneous channel opening likely resulted from immobilization of the receptor in the closed channel conformation. However, a distinguishing insight from this work is that conformational arrest by α-Btx impairs the ability of the remaining unoccupied sites to either bind agonist or couple binding to channel opening. Analogously, a conformational arrest mechanism may explain competitive antagonism by methyllycaconitine (MLA). Following MLA antagonism of α7 and washout of the drug, recovery of agonist-induced macroscopic currents showed a sigmoid time course best explained by requiring MLA dissociation from all five sites before agonist-induced activation could occur^31^. Thus although MLA and α-Btx interact very differently at the α7-binding site, binding of a single MLA molecule appeared enough to inhibit α7.

While our results show that a single bound α-Btx molecule greatly suppresses channel opening, it is important to consider the extent of suppression. For the concentration of α-Btx used in the present experiments (50 nM, ∼20 times the K_d_), at equilibrium, occupancy by α-Btx is expected to be 95%. Thus, if a single α-Btx was enough to fully block channel opening, for receptors with a single α-Btx-sensitive subunit, the maximum suppression of channel opening is expected to be ∼95%. For the 1:4 transfection ratio, the frequency of openings from receptors with a single α-Btx-sensitive subunit is reduced by more than 85% and for the 1:3 transfection ratio, the reduction approaches 100%. These values bracket the expected suppression of 95% and may reflect limitations of sampling. Thus, we favour the interpretation that occupancy by a single α-Btx is enough to fully block channel opening.

**Figure 7:**
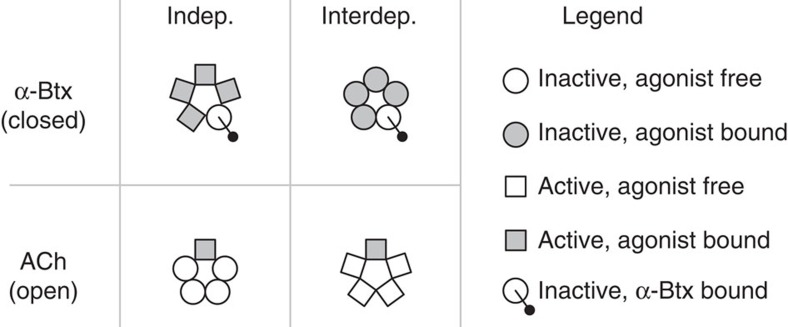
Scenarios for conformational coupling amongst subunits in α7. In an independent scenario (left column), individual subunits can adopt different conformations irrespective of the other subunits within the pentamer, whereas in the interdependent scenario (right column), the conformations of individual subunits are coupled to each other, maintaining subunit conformational symmetry in the entire pentamer. In the situation where a single α-Btx is bound to α7, the channel is closed (top row), while in the absence of α-Btx, when a single acetylcholine (ACh) is bound, the channel is open (bottom row). The legend on the right explains the different shapes used to represent active (squares) and inactive (circles) conformations of each subunit, as well as their occupancy with either agonist (grey shading) or α-Btx (black ball and stick).

Given this interpretation, the ability of a single α-Btx molecule to block agonist-induced channel opening distinguishes independent from interdependent conformational changes of the α7 subunits (Fig. 7). In the independent mechanism, an individual subunit can change conformation regardless of the conformations of the other subunits and agonist occupancy of a single binding site opens the channel regardless of the conformations of the remaining subunits. However, given that a single α-Btx molecule blocks agonist-induced channel opening, we can discount the independent mechanism, as it incorrectly predicts that binding of five α-Btx molecules would be required.

By contrast, our findings are compatible with an interdependent mechanism, where a single agonist occupancy opens the α7 channel, but only when all five subunits can adopt an active conformation (Fig. 7). In the interdependent mechanism, occupancy by a single α-Btx molecule arrests all five subunits in the inactive conformation, and the energy gain from binding multiple agonist molecules is not enough to overcome conformational arrest by α-Btx. Yet, in the absence of α-Btx, occupancy by a single agonist is enough to produce the activated conformation of all five α7 subunits. Clearly the energy landscape of α7 is exquisitely poised to take advantage of modest changes in energy from occupancy by a single agonist. At the same time, α-Btx exploits the fact that the energy gained from agonist binding is modest compared with conformational arrest imposed by a single bound toxin.

In early models of protein allostery, a key postulate was that on activation, individual subunits in an oligomer maintain conformational symmetry^32^. Maintenance of symmetry predicts that arresting a single site in an inactive conformation will thwart activation of the entire oligomer, which is consistent with a single α-Btx-sensitive subunit imparting toxin-sensitivity to α7. Although we cannot distinguish whether subunit conformational changes occur in a sequential^33^ or concerted^32^ fashion, our results suggest that in the open channel state, interdependent conformational changes maintain symmetry of the five α7 subunits, which allows a single bound α-Btx to block channel opening.

## Methods

### Materials

[^125^I]-labelled α-Btx was from Perkin Elmer, while unlabelled α-Btx and 1-(5-Chloro-2,4-dimethoxy-phenyl)-3-(5-methyl-isoxazol-3-yl)-urea, known as PNU-120596, were from Tocris Biosciences. All other chemicals, including acetylcholine chloride (ACh) and nicotine (Nic), were from Sigma-Aldrich.

### Site-directed mutagenesis

Mutant α7 cDNAs were constructed using the QuikChange Site-Directed Mutagenesis kit (Stratagene) and then confirmed by sequencing the entire coding region. On the basis of previous findings with a chimeric α7/5-HT_3A_ receptor^23^, the α-Btx-resistant mutant contains two substitutions within the α7 ligand-binding domain (F187K and E189N; Fig. 1). The low conductance form of α7 (WT_LC_) results from substitution of three arginines into the α7 cytoplasmic domain (Q428R, E432R and S436R). Arginines at these positions are responsible for the small unitary conductance of the homologous 5-HT_3A_ receptor^26^. In the absence of calcium at −70 mV, these arginine substitutions reduce the amplitude of α7 from ∼11 to ∼4 pA (Figs 4, 5), and do not alter α7-activation kinetics or sensitivity to α-Btx. The sequences of all mutagenic primers are provided in Supplementary Table 1.

### Expression of human α7

BOSC 23 cells, modified HEK 293 cells^34^, were transfected by calcium phosphate precipitation. Expression of human α7 (GenBank accession number X70297) in mammalian cell lines requires co-transfection with the intracellular chaperone Ric-3 (refs 35, 36). As in previous studies^17^,^25^, Ric-3 and α7 DNAs were co-transfected at a ratio of 12:1 (w/w), with a total of 0.25 μg α7 DNA for a 35-mm culture dish. All subunit mixing experiments were performed by co-transfecting cDNA encoding α-Btx-resistant mutant, normal conductance subunits with that encoding wild-type, low conductance subunits (WT_LC_) at a weight-to-weight ratio of either 1:4 or 1:3 (mutant:WT_LC_). Transfections were carried out for between 7 and 12 h in DMEM with 10% fetal bovine serum, and terminated by exchanging the medium. Cells were used for single-channel recordings 0.5–3 days post transfection. To facilitate identification of transfected cells, a separate plasmid encoding green fluorescent protein was included in all transfections. To minimize the effects of day-to-day variability in the level of expression, all experiments with and without α-Btx were performed in parallel, on the same day and with cells transfected with the same transfection mixture.

### Single-channel recordings

Recordings from transfected BOSC 23 cells were obtained in the cell-attached patch configuration^37^ at a membrane potential of –70 mV and at 20 °C (refs 25, 28, 38). To ensure that the applied potential was the same for all experiments, any ‘pipette offset’ was manually zeroed before patch formation. The bath solution contained (in mM): 142 KCl, 5.4 NaCl, 0.2 CaCl_2_ and 10 HEPES, pH adjusted to 7.4 with KOH. All pipette solutions contained (in mM): 80 KF, 20 KCl, 40 K-Aspartate, 2 MgCl_2_, 1 EGTA and 10 HEPES, with the pH adjusted to 7.4 with KOH. For the experiments in Figs 4, 5, 6, the pipette solution also included 100 μM ACh and 1 μM PNU. ACh and PNU were added directly to pipette solutions from 1000 × concentrated stock solutions of ACh in pipette solutions, and PNU in dimethylsulphoxide. Pipette solutions were stored in aliquots at –80 °C until immediately before each experiment. Patch pipettes were pulled from glass capillary tubes (No. 7052, King Precision Glass) and coated with Sylgard (Dow Corning). Single-channel currents were recorded and low-pass filtered at 10 kHz using an Axopatch 200 B patch-clamp amplifier (Molecular Devices), and digitized at 20 μs intervals (that is, 50 kHz) with an InstruTECH ITC-16 interface (Heka Elektronik).

For experiments in which nicotine was added to the bath solution (Fig. 2), cells were bathed in 0.7 ml of bath solution , and a GΩ seal was formed in the cell-attached patch configuration. The patch pipette contained 10 μM PNU in pipette solutions . After 5 min of recording, 0.3 ml of 333 μM Nicotine in bath solution was added drop wise to the external solution surrounding the patch ([nicotine]_final_=100 μM). For all α-Btx experiments, cells in 35-mm culture dishes were aspirated of their surrounding media and gently washed with 1.0 ml of bath solution containing 50 nM α-Btx. This wash solution was then replaced with fresh 50 nM α-Btx in bath solution, and the cells were incubated for a minimum of 20 min before forming a GΩ seal in the cell-attached configuration.

Because stretches of continuous recording lasting several minutes are shown, electrical artifacts inherent to the patch clamp head stage are inevitable (that is, head stage capacitor resets). The patch clamp electronics were tuned so that these artifacts were both minimized and recognizable as brief (∼1 ms) negative baseline deflections, which allowed them to be identified and subsequently removed for presentation purposes. The data were analysed before removal of these artifacts.

### Single-channel analysis

Single-channel analysis was performed using the program TAC 4.2.0 (Bruxton), which digitally filters the data (Gaussian response), interpolates the digitized points using a cubic spline function and detects events using the half-amplitude threshold criterion^39^. Electrical fingerprinting experiments were performed as in previous studies^17^, except in the present work, rather than detecting the durations of openings, we were simply interested in whether channels with a specified number of bound α-Btx molecules could open. We therefore did not perform dwell time analysis, and instead focused solely on the amplitudes of the observed openings. All analysis was carried out after digitally filtering the data at 1 kHz, which greatly simplifies the assignment of single-channel amplitudes. Only the first 20 min of each recording was analysed.

Due to inherent rapid desensitization, α7 has unusually brief openings (<1 ms), many of which do not reach full amplitude owing to filter bandwidth limitations. We therefore used PNU, which slows the onset of α7 desensitization^24^, to trap activated α7 channels in an open conformation. Clusters of PNU-potentiated openings last from seconds to minutes^25^,^40^, facilitating their identification and allowing for accurate registering of their amplitudes. Only clusters of openings longer than 1 s, and preceded or followed by a stable baseline longer than 250 ms, were included in the analysis of amplitudes. Each cluster of openings was grouped, and a single amplitude assigned to the entire cluster. For each cluster, a baseline current and an open channel current were assigned by fitting all-points histograms with a Gaussian component for the baseline, and another Gaussian component for the open channel current. The difference between the open channel current and the baseline current defined the amplitude of each cluster.

Given that PNU potentiated openings last from seconds to minutes, patches with multiple channels frequently gave rise to clusters of openings superimposed on one another. In such cases, following the above criteria, the amplitude of only a single cluster, either at the beginning or the end of the superimposed openings, was counted. In instances where such episodes began with a cluster of one amplitude and ended with a cluster of an unambiguously different amplitude (that is, Figs 4, 5), both clusters were counted, as long as they were at least 1 s long, and preceded or followed by 250 ms of stable baseline. While it is theoretically possible to measure the individual amplitudes of channels superimposed on one another, this was not done because the open channel noise envelope is substantially larger than that of the baseline noise, which leads to increasing uncertainty in amplitude estimates for channels in successive layers of superimposed openings. For this reason, all amplitude measurements were derived solely from clusters neighbouring stable baseline.

For all conditions, clusters of openings from between 7 and 21 different recordings were pooled, and event-based scatter plots and amplitude histograms constructed (Figs 4, 6). To determine the amplitudes of α-Btx-resistant normal conductance homopentamers mutant, and wild-type, low conductance homopentamers (WT_LC_), their corresponding amplitude histograms were each fit with a single Gaussian component, whose maxima and width defined the mean±s.d. (1σ) of their respective amplitude class (Fig. 4). To determine the amplitudes of hetero-pentamers comprised of mutant and WT_LC_ subunits, an amplitude histogram was constructed by pooling the measured amplitudes from all subunit mixing experiments (both 1:3 and 1:4 cDNA ratios; mutant:WT_LC_). Gaussian components corresponding to mutant and WT_LC_ homopentamers were inserted, and then the remaining amplitude classes were distinguished and fit by a sum of additional Gaussian components (Figs 4, 6), where once again the maxima and width of each component defined the mean±s.d. (1σ) of each amplitude class (Fig. 4). Once amplitude classes had been established, a minimum number of components from the predetermined amplitude classes were fit to histograms from the α-Btx incubation experiments. The proportion of each component was then allowed to vary to improve the fit (Fig. 6). Throughout this fitting procedure the width of each component was kept constant, except in Fig. 6a, where the component corresponding to channels with 4mutant+1WT_LC_ subunits was fit with a slightly narrower Gaussian.

For the subunit mixing experiments with a 1:4 ratio (mutant:WT_LC_) of cDNAs, patches without incubation with α-Btx were typically very active, with multiple superimposed openings (Fig. 5). However, after incubation with α-Btx, more than half of the patches from cells expressing this same 1:4 ratio were devoid of activity (that is, 13 of 21 patches). To be counted as a patch devoid of activity (that is, any of the 13 patches) the recording had to remain stable for at least 10 min, and not display any discernible α7 openings (that is, no non-potentiated or PNU-potentiated openings).

The dramatic reduction in patch activity for cells incubated with α-Btx is an important aspect of the present data. Because event-based amplitude histograms only contain information on the absolute number of events and not their frequency, histograms, with and without α-Btx incubation, were expressed relative to both the total number of observed events and the total duration of all recordings for each condition (Fig. 6). The number of clusters neighbouring baseline that were included in initial event-based amplitude histograms was divided by the total recording time (in minutes), which resulted in an estimate of the frequency of baseline neighbouring clusters per minute. However, because patches from cells that were not incubated with α-Btx consistently displayed many levels of superimposed openings (Fig. 5), the frequency of baseline clusters per minute is a gross underestimate of the actual patch activity. To rectify this, the total number of openings, regardless of amplitude, was determined by following the same criteria above (that is, cluster longer than 1 s and separated by at least 250 ms), except the requirement that clusters neighbour baseline was relaxed. This allowed for estimates of the total number of clusters in each condition, including those superimposed on one another, which was then divided by the number of baseline clusters included in the initial event-based amplitude histograms. The frequency of baseline clusters (that is, the first term below) was then multiplied by this ratio as follows:




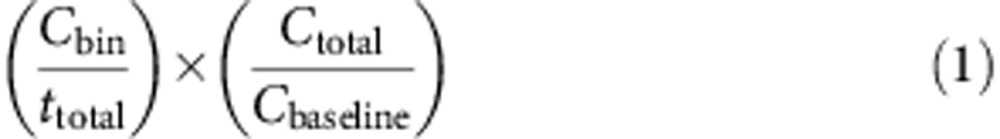




Where ‘*C*_bin_’ is the number of baseline clusters contained in each bin of the initial event-based amplitude histograms; ‘*t*_total_’ is the combined time, in minutes, for all recordings for a given condition; ‘*C*_total_’ is the combined total number of observed clusters, including those superimposed on one another, for each condition; and ‘*C*_baseline_’ is the combined number of all clusters neighbouring baseline, for each condition. The resulting amplitude histogram gives a measure of the frequency of events with specified amplitudes, and allows for comparisons of cluster frequency across conditions (that is, with and without α-Btx incubation). A tacit assumption is that channels in the patch open independently of one another and therefore the amplitude distribution derived from sampling events at baseline approximates the amplitude distribution if it were possible to accurately measure the amplitudes of all openings, including those that are superimposed.

### [^125^I]-α-Btx binding experiments

Steady state [^125^I]-α-Btx binding experiments were carried out at room temperature (∼21 **°**C). Transfected cells were collected by gentle agitation in Dulbecco’s phosphate-buffered saline (Gibco) with 5 mM EDTA (pH 7.4), centrifuged at 1000*g* for 1 min and resuspended in potassium Ringer’s solution (in mM): 140 KCl, 5.4 NaCl, 1.8 CaCl_2_, 1.7 MgCl_2_, 25 HEPES and 30 mg l^−1^ bovine serum albumin, pH adjusted to 7.4 with KOH. Collected cells were combined and divided into equal aliquots corresponding to approximately one quarter of the cells in a 10-cm tissue culture dish. Individual aliquots were incubated with increasing concentrations (0–100 nM) of [^125^I]-labelled α-Btx for 1 h and then filtered to Brandel GF-B filter papers using a Brandel M48-T Cell Harvester. Unbound toxin was removed by successive washes with 140 mM NaCl in 10 mM HEPES, pH 7.4. Deposited cells, with bound [^125^I]-α-Btx, were then counted for 2 min in a Perkin Elmer 1470 automatic gamma counter. To ensure that measurements from cells transfected with different cDNAs were comparable, all experiments were performed on the same day, with the same batch and thus specific activity of [^125^I]-α-Btx. Total and non-specific binding were globally fit using the GraphPad Prism 5.0 software (Fig. 3), where non-specific binding was determined by measuring [^125^I]-α-Btx binding to cells transfected with the human-muscle β1 subunit. Specific binding is the difference between total and non-specific binding.

### Kinetics of [^125^I]-α-Btx dissociation

To measure the time course of [^125^I]-α-Btx dissociation (Fig. 3), a cell suspension, prepared as described above, was mixed with 50 nM [^125^I]-α-Btx and incubated for 1 h at 21 **°**C. The suspension was then centrifuged at 1000*g* for 1 min, the supernatant was removed and the cells were resuspended in 20 ml of potassium Ringer’s solution. Aliquots of the cell suspension were then rapidly filtered through type A/E glass fibre filters (Gelman Sciences) at specified times after resuspension. Unbound toxin was removed by successive washes with 140 mM NaCl in 10 mM HEPES, pH 7.4. To determine non-specific binding, the same procedure was applied to cells transfected with the human muscle AChR β1-subunit cDNA. After subtracting non-specific binding, a single exponential decay was fitted to the data using the GraphPad Prism 5.0 software.

## Additional information

**How to cite this article:** daCosta, C. J. B. *et al*. Stoichiometry for α-bungarotoxin block of α7 acetylcholine receptors. *Nat. Commun.* 6:8057 doi: 10.1038/ncomms9057 (2015).

## Supplementary Material

Supplementary Information Supplementary Table 1
