# Supplementary material for: Stoichiometry for α-bungarotoxin block of α7 acetylcholine receptors
Source: Nat Commun. 2015 Aug 18;6:8057. doi: 10.1038/ncomms9057 (PMC4544739; doi:10.1038/ncomms9057)
Supplement: Supplementary Information — Supplementary Table 1 [file ncomms9057-s1.pdf]

Supplementary Table 1: Oligonucleotide sequences for mutagenic primers

(1) α-Btx-resistant α7 mutant (F187K + E189N)

α7 (F187K + E189N)		
5' → 3'	=	5'-GGCAAGAGGAGTGAAAGGAAGTATAACTGCTGCAAAGAGCC-3'
3' → 5'	=	5'-GGCTCTTTGCAGCAGTTATACTTCCTTTCACTCCTCTTGCC-3'

(2) α7, low conductance subunit (Q428R + E432R + S436R)

Step 1: α7 (Q428R)		
α7 (Q428R)		
5' → 3'	=	5'-GCTTCCGCTGCCGGGACGAAAGCGAGG-3'
3' → 5'	=	5'-CCTCGCTTTCGTCCCGGCAGCGGAAGC-3'
Step 2: α7 (S436R) + (Q428R)		
α7 (S436R)		
5' → 3'	=	5'-GGCGGTCTGCAGAGAGTGGAAGTTCG-3'
3' → 5'	=	5'-CGAACTTCCACTCTCTGCAGACCGCC-3'
Step 3: α7 (E432R) + (Q428R + S436R)		
α7 (E432R)		
5' → 3'	=	5'-CGGGACGAAAGCAGGGCGGTCTGCAGAGAGTGG-3'
3' → 5'	=	5'-CCACTCTCTGCAGACCGCCCTGCTTTCGTCCCG-3'
